# *Bartonella henselae* Antibodies after Cat Bite^1^

**DOI:** 10.3201/eid1412.080002

**Published:** 2008-12

**Authors:** Katarina Westling, Anna Farra, Christina Jorup, Åsa Nordenberg, Bo Settergren, Eva Hjelm

**Affiliations:** Karolinska Institutet, Stockholm, Sweden (K. Westling, A. Farra, C. Jorup, B. Settergren); University Hospital, Uppsala, Sweden (Å. Nordenberg, E. Hjelm); 2 Current affiliation: Central Hospital, Kristianstad, Sweden.

**Keywords:** Bartonella henselae, seroconversion, transmission pathways, cat bite, Bartonella, letter

**To the Editor:** *Bartonella henselae* is the causative agent of cat-scratch disease, which is the most common form of human bartonellosis (*1*). In immunocompromised patients, e.g., HIV-infected patients, *B. henselae* can give rise to longstanding fever, bacillary angiomatosis, and peliosis hepatitis (*2*). Domestic cats are the reservoir for *B. henselae*, and cat fleas transmit the organism between cats (*3*). The seroprevalence and culture findings of *Bartonella* spp. in cats have been shown to be low in Sweden (*4*,*5*) compared with warmer areas (*6*). Cat-scratch disease is most often spread from cats to humans by scratches, but other forms of transmission, including cat bites, have been suggested (*7*).

To determine seroprevalence of antibodies against *B. henselae* in Sweden, we used data from a recently published prospective study of patients with infected cat bites (*8*). In addition to the information about bites, information about cat scratches was collected by retrospective review of the patients’ medical records. Serum samples were taken during the patient’s first visit to a hospital and at a follow-up visit about 2 weeks later. The study was approved by the local ethics committee.

Immunoglobulin G against specific *Bartonella* spp. was detected by the immunofluorescence antibody test (*1*). Cell-cultivated antigens were prepared from the following strains: *B. henselae* Houston-1 (ATCC 49882), *B. henselae* Marseille (CIP 104756), *B. henselae* Berlin1 (M. Arvand), *B. henselae* K68 (E. Olsson-Engvall), *B. elizabethae (*ATCC 49927*)*, and *B. grahamii* (ATCC 700132). The cutoff values for a positive immunofluorescence antibody test result were chosen as >128 for *B. henselae* and *B. grahamii* and >256 for *B. elizabethae*. The titers are expressed as the reciprocal of the end-point dilution. For controls, we also analyzed serum from 117 blood donors with these antigens. The χ^2^ test was used for statistical analysis.

We analyzed antibodies to *Bartonella* spp. in serum from 71 patients (51 women and 20 men), median age 47 years (range 15–85 years). Only 11 patients had fever. Cat scratches were reported for 17 patients. A single serum sample was obtained from 37 of the 71 patients, and an additional convalescent-phase sample was obtained from 34 patients after a median of 16 days (range 6–54 days).

Antibodies against any *B. henselae* strain were found for 24/71 (34%) patients, against *B. elizabethae* for 9/71 (13%), and against *B. grahamii* for 12/71 (17%). A total of 13/71 (18%) patients showed reactivity to *B. henselae* only*.* Antibodies to any *Bartonella* spp. were found for 28/71 (39%) of the patients. As many as 13/24 (54%) serum samples with antibodies against *B. henselae* reacted to antigens of only that species. More patients (19/71; 27%) reacted to the antigen from the cat in Sweden, K68, than to other strains. The least common reactivity found in this study was against the *B. henselae* Marseille strain.

Of the 117 controls, 1 (0.8%) had antibodies against *B. henselae* K68 antigens, 3 (2.6%) against Berlin1, 2 (1.7%) against Marseille, 1 (0.8%) against Houston-1, and 4 (3.4%) against any *Bartonella* spp. The difference between patients and controls was significant (p<0.001).

Seroconversion was reported for 6 of the 34 patients (18%) from which 2 serum samples were analyzed (Table). Among those who seroconverted for *B. henselae,* 1 had fever and only 2 reported having been scratched. Two of the patients who seroconverted were treated with doxycycline, and 1 was treated with ciprofloxacin. In addition, 1 patient with Sjogren disease was initially treated with penicillin, and later a hemangioma-like exanthema developed. Because of severe acne, the patient was treated with doxycycline for 6 months. The other patients who seroconverted were treated with penicillin or amoxicillin.

**Table:** Titers against *Bartonella* spp. antigens in 6 patients who seroconverted

Patient sex/ age, y	Reciprocal titer		Interval, d	Antigen	High titer to other antigen
	Acute-phase serum	Convalescent-phase serum			
F/77	32	128	6	*B. henselae* Berlin1	*B. henselae* K68
F/50	32	256	17	*B. henselae* Berlin1	0
M/74	128	512	19	*B. grahamii*	*B. henselae* K68
F/62	32	128	44	*B. elizabethae*	*B. henselae* K68
M/56	32	128	15	*B. henselae* Houston-1	*B. grahamii*
F/23	32	128	10	*B. henselae* Berlin1	*B. grahamii*

Seroconversion for *B. henselae* occurred in 4 patients, of which only 2 had reported a scratch. Three of these patients reacted to *B. henselae* Berlin1, and 1 reacted to the Houston-1 strain. Because symptoms did not differ between the patients who did seroconvert and those who did not, these findings could indicate subclinical infection.

The prevalence of immunoglobulin G against *B. henselae* in particular was shown to be much higher than that previously reported in Sweden (*9*). Earlier studies used only 2 *B. henselae* antigens (Houston-1 and Marseille) compared with the 4 different *B. henselae* antigens used in the present study. Most reactivity to *B.* *henselae* in the present study was directed against the Swedish isolate K68 (27%); only 0.8% of controls had antibodies against that antigen.

An increased prevalence of antibodies against *B. henselae* after exposure to cats has been reported from Spain (*10*). Because seroconversion against *B*. *henselae* occurred in 2 patients who had not been scratched, cat bites may contribute to transmission of *B. henselae*.
